# Comparative effectiveness of 3 Traditional Chinese Medicine treatment methods for idiopathic pulmonary fibrosis: A systematic review and network meta-analysis protocol

**DOI:** 10.1097/MD.0000000000016325

**Published:** 2019-07-26

**Authors:** Li-Jian Pang, Jian-Ping Liu, Xiao-Dong Lv

**Affiliations:** a Beijing University of Chinese Medicine, Beijing; b Liaoning University of Traditional Chinese Medicine; c Affiliated Hospital of Liaoning University of Traditional Chinese Medicine, Shenyang; d Center for Evidence Based Chinese Medicine, Beijing University of Chinese Medicine, Beijing, China.

**Keywords:** Chinese herb formula, idiopathic pulmonary fibrosis, protocol, systematic review

## Abstract

**Introduction:**

The morbidity of idiopathic pulmonary fibrosis (IPF) was found in an increasing trend, progressive worsening of symptoms and deterioration in lung function tend to trigger off a lower quality of life (QoL). Only pirfenidone and nintedanib have been recommended in the guidelines, which can modify the disease process. However, no evidence was verified to significantly alleviate the main clinical manifestations of IPF. At present, Chinese herbal formula (CHF) is widely prescribed as an adjunct to western medicine to treat the disease, and have shown promising benefits on clinical symptoms and QoL. There are mainly 3 Traditional Chinese Medicine (TCM) treatment methods guiding the composition of CHFs, which are devoting to comfort the common symptoms of IPF. Nevertheless, the paucity of direct comparative evidence of them posed a challenge for clinicians to determine the relative merits options. Therefore, we formulate this protocol, which is described for a systematic review to investigate relative advantages among different TCM treatment method and provide more reliable evidence for clinical decision-making.

**Methods and analysis:**

A systematic literature search will be employed in 10 electronic databases. Inclusion criteria are randomized control trials of CHFs composed based on the 3 TCM treatment methods, which act as an adjuvant treatment with routine drugs, compared with routine drugs alone. The primary outcomes we focus on include St George's Hospital Respiratory Questionnaire (SGRQ) scores, TCM symptom (dyspnea, cough) scores. The research screening, data extraction, and methodological quality assessment will be conducted by 2 individuals separately, and dispute will be adjudicated by a third senior reviewer. We will employ network meta-analysis (NMA) in a Bayesian framework with vague priors and the surface under the cumulative ranking curve (SUCRA) to obtain the comprehensive rank for the 3 TCM treatment methods.

**Results:**

This systematic review will provide an evidence of CHFs composed under the guidance by 3 TCM treatment methods with routine drugs, compared with routine drugs alone for IPF, and will submit to a peer-reviewed journal for publication.

**Conclusion:**

The conclusion of this systematic review will provide evidence for relative advantages among the 3 TCM treatment methods.

## 1 Introduction

Idiopathic pulmonary fibrosis (IPF) is a chronic and progressive lung disease, which is classed as an special type of idiopathic interstitial pneumonia (IIP). It is known to affect males more than females and in particular middle age.^[1]^ Overall, the morbidity of IPF was found in an increasing trend year by year,^[2–5]^ and epidemiological documents suggested that its incidence is 6.8 to 16.3 per 100,000.^[6]^ The etiology of IPF is still unknown. Excessive smoking is significantly associated with the pathogenesis of IPF, in addition, environmental exposure, gastroesophageal reflux, genetic factors, and microbial infection may be somewhat related with the disease.^[7]^ The prognosis of individuals with IPF is poor,^[8]^ and the median survival time is generally estimated to be 2 to 5 years from diagnosis.^[7,9]^

Dyspnea and cough are the most common symptoms in cases with IPF, and breathless have been found to be associated with mortality.^[10]^ A retrospective study showed that more than 90% of patients had symptoms of breathless and 60% with cough.^[11]^ progressive worsening of symptoms and deterioration in lung function can trigger off a gradual limitation of physical activity, and the suffers become more debilitated, which can lead to a lower quality of life (QoL).^[10,12,13]^ St George's Hospital Respiratory Questionnaire (SGRQ) is a measure designed to assess the impact of obstructive airway disease on overall health, daily life and perceived well-being, which appears to possess reasonable validity and reliability in IPF.^[14]^

Therefore, therapeutic strategies for IPF ought to be comprehensive. Alleviating symptoms, improving QoL and delaying progression of disease are equally important for patients. Available options of modern drugs are limited currently, only pirfenidone and nintedanib are recommended in the guidelines, which can modify the disease process.^[2]^ However, they have not been verified to alleviate the major symptoms in IPF.^[15]^ Accordingly, some gaps and deficiencies remain to be further filled in routine pharmacotherapy for IPF, such as relief of main manifestations and improvement of QoL.

Traditional Chinese medicine (TCM) has been applied more than thousands of years for the treatment of respiratory disease in China and some other Asian counties. Although TCM is not the mainstream for treating IPF, it has become increasingly accepted as a form of complementary or complementary medicine in western countries.^[16]^ Chinese herb formula (CHF), as an important component of TCM therapeutic measures, is widely prescribed as an adjunct to western medicine to treat IPF in clinical guideline.^[17]^

At present, there are mainly 3 TCM treatment methods guiding the composition of CHFs to relieve main symptoms for treating the cases, which are “activating blood circulation and removing blood stasis” method, “benefiting Qi and activating blood circulation” method, and “dredging collaterals” method. Several systematic reviews synthesized the effectiveness of CHFs composed under direct of the single TCM treatment methods, which showed that CHFs combined with routine pharmacotherapy has promising benefits on QoL and clinical symptoms when compared with routine pharmacotherapy alone.^[18–20]^ Nevertheless, the paucity of direct comparison evidence among these TCM treatment methods posed a challenge for clinicians to determine the relative merits options.

Compared with the pairwise meta-analysis, network meta-analysis (NMA) can compare and rank the effectiveness of 3 or more measures for the treatment of a disease, which is important for clinicians to make the best option.^[21–22]^ Therefore, this systematic review aims to synthesize indirect comparative evidences of the CHFs treating for IPF composed based on the 3 TCM treatment methods, which act as an adjuvant therapy with conventional drugs, then to explore the relative strengths of them, and provide more reliable evidence for clinical decision-making.

## 2 Methods

### 2.1 Registration

The study protocol has been registered on international prospective register of systematic review (PROSPERO registration number: CRD42018094920). The procedure of this protocol will be conducted according to the Preferred Reporting Item for Systematic Review and Meta-analysis Protocols (PRISMA-P) guidance.^[23]^

### 2.2 Inclusion and exclusion criteria

#### 2.2.1 Type of study

Randomized controlled trials (RCTs) are eligible for inclusion, which should evaluate at least 1 primary outcome. Quasi-RCTs, duplicated publications, narrative publications, case reports, editorials, animal researches, and pharmacological experiments will be excluded.

#### 2.2.2 Participants

The participants should be diagnosed with IPF by using clearly defined or internationally recognized criteria, and aged at least 18 years old. The cases with following diseases will not meet selection criteria:

(1) Respiratory disease like asthma, bronchiectasia, chronic obstructive pulmonary disease, and so on.
(2) Severe liver, kidney, heart disease, and so on.
(3) Patients were in the acute exacerbation period of IPF (AE-IPF).

### 2.3 Interventions and comparators

Interventions include CHFs composed under the guidance by 1 of the 3 TCM treatment methods combined with conventional pharmacotherapy (pirfenidone, nintedanib, and N-acetylcysteine) will meet the eligibility criteria. The form, dosage, frequency, and duration of treatment will not be limited. The same conventional pharmacotherapy must be used in the comparator arm.

In view of the low price and good tolerance, N-acetylcysteine (NAC) is still commonly used by patients on the basis of clinical opinion in China,^[24,25]^ though have not been recommended in the guideline, we tend to include it as one of conventional pharmacotherapy.

### 2.4 Outcome measures

The primary outcomes include:

(1) SGRQ scores,
(2) TCM symptom scores (dyspnoea, cough).^[26,27]^

The secondary outcomes are:

(1) change in forced vital capacity (FVC),
(2) total clinical efficacy rate,
(3) acceptability (discontinuation due to any adverse events during treatment),
(4) acute exacerbation (AE),
(5) all-cause mortality,
(6) IPF-related mortality.

### 2.5 Search strategy

We will perform an all-round search for published studies in 10 electronic databases from their inception to December 2018: PubMed, EMBASE (include MEDLINE), Cochrane Central Register of Controlled Trials (CENTRAL), Ovid (Journals @ Ovid Full Text and Ovid MEDLINE (R)),CINAHL, AMED, Chinese Biomedical Database (CBM), Chinese National Knowledge Infrastructure (CNKI), Chongqing VIP information (CQVIP), and Wanfang database. The following sources will also be searched to identify clinical trials which are in progress or completed: Current Controlled Trials, WHO clinical trials registry, Clinical Trials.gov trials registry, The Australian New Zealand Clinical Trials Registry, Centre Watch, and Chinese Clinical Trial Registry. If any, we will try to contact the correspondence author to obtain the data we need. The references of systematic reviews and literature included will also be checked. The search strategies for selecting the fields of title, abstract or keyword will be adjusted according to different characteristics of databases. The language is limited to Chinese and English. The search terms are shown in Table 1.

**Table 1:**
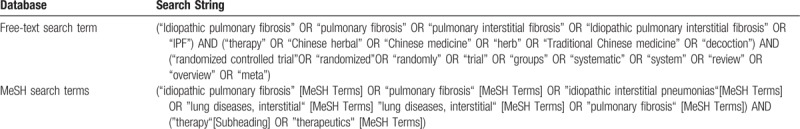
The search terms.

### 2.6 Study selection

**Figure 1:**
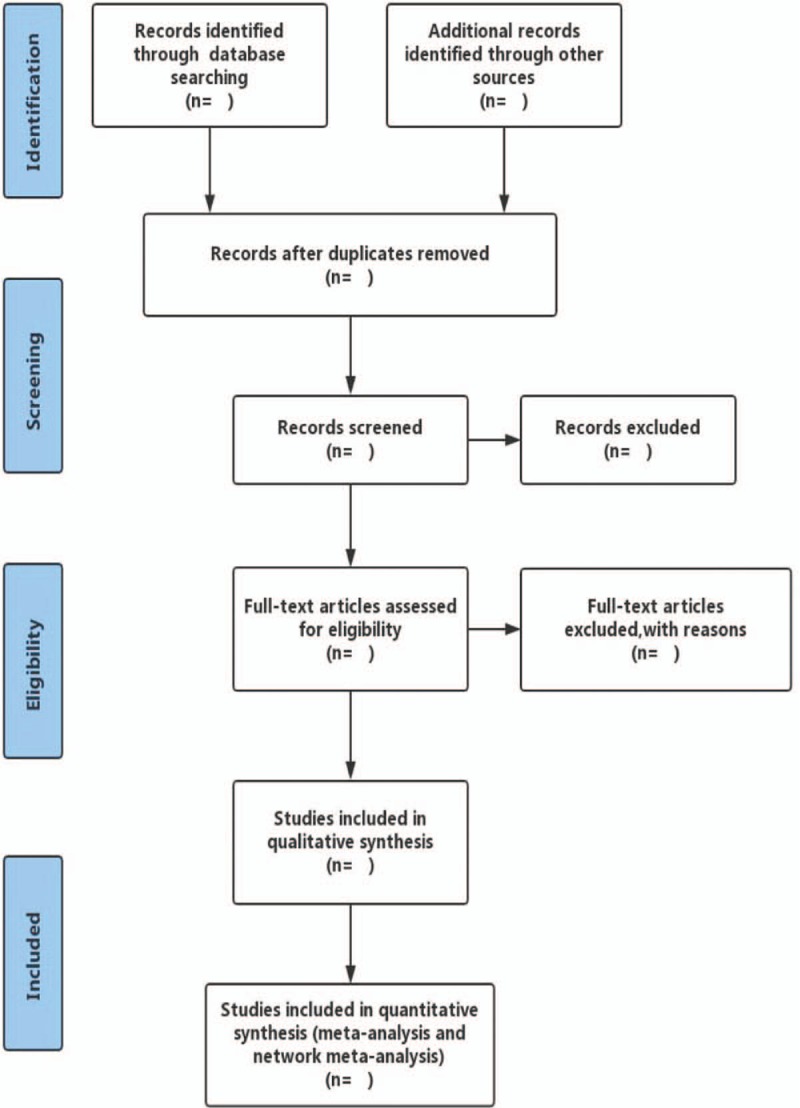
The primary selection process legend: details of the selection process will be presented in the PRISMA flow chart.

We will use NoteExpress V3.0 software to manage the studies retrieved and remove duplications. Two independent reviewers (PLJ and LXD) will evaluate title and abstract of studies. The further screening will be conducted to review the full papers to select eligible studies by the same 2 independent reviewers. Final decisions on exclusion at both the abstract and full-text review stages will be made by consensus process and any disagreements will be judged by a third senior reviewer (LJP). The reviewers will record all studies that do not meet the inclusion criteria and provide the rationale for their exclusion. Details of the selection process will be presented in the PRISMA flow chart. (Fig. 1)

### 2.7 Data extraction

Data extraction will undergo a process with independent abstraction by the 2 reviewers (PLJ and LXD), the final decisions will be made by consensus process and disagreements will be resolved by a third senior reviewer (LJP). We will use Epidata 3.1 software (The EpiData Association, Odense, Denmark, 2003–2008) to extract data and check the consistency of information. Extracted data will include first author, publication year, participants (age, sex, number of subjects, course of disease), study design (randomization, allocation concealment, blinding, and etc), interventions (frequency and duration), controls (frequency and duration), outcomes. If required information is not reported, we will try to request it from the corresponding author of the studies.

### 2.8 Risk of bias assessment

Two researchers (PLJ and LXD) will assess methodological quality of included studies separately by the Cochrane collaboration's risk of bias tool and reported on a per-study basis.^[28]^ We will consider the following:

(1) random sequence generation (selection bias)
(2) allocation concealment (selection bias)
(3) blinding of participants and personnel (performance bias)
(4) blinding of outcome assessment (detection bias)
(5) incomplete outcome data (attrition bias)
(6) selective reporting (reporting bias)
(7) other sources of bias (other bias).

For each domain, we will make “yes (low bias)”, “no (high bias)” and “not clear (lack of information or bias uncertainty)” judgment to evaluate the risk of bias (RoB) in each of the studies. Consensus will be reached by discussion with a third party (LJP) in case of discrepancy.

### 2.9 Dealing with missing data

In the event of missing data exist in included research; we will contact the corresponding authors. In case of missing data are unobtainable, intention-to-treat (ITT) analysis will be performed, if possible, and sensitivity analysis will also be conducted to address the potential impact of missing data,^[29,30]^ which will be discussed if necessary.

### 2.10 Statistical analysis

#### 2.10.1 Pairwise meta-analysis

For each outcome, we will employ the conventional meta-analysis by Revman 5.3 software (Cochrane Collaboration, Oxford, UK). If available data are insufficient, qualitative synthesis will be performed. Dichotomous data will be presented as odds ratio (OR) with corresponding 95% confidence intervals (CIs) expect survival outcomes, which will be reported as hazard ratio (HR), because it is more informative than OR and account for the time to death. The decline in FVC may be measured as an absolute change from baseline (liters) or a decline in percent predicted. We will use the standardized mean difference (SMD) approach to convert these measurements to a common scale,^[31]^ and vital capacity will be assumed to be the same as FVC in IPF. The other continuous variables will be calculated as mean difference (MD) with corresponding 95% CIs. Statistical heterogeneity will be quantified by the I^2^ value for each pairwise comparison that is informed by at least 2 trials, If I^2^ <50%, we will use fixed-effects (FE) model, if I^2^ ≥50%, which indicate significant heterogeneity, random-effects (RE) model will be adopted. Where heterogeneity is detected, accepted methods will be used to explore based on specified effect modifiers as follows: participant's average age, publication year, sample size, dosage form, treatment duration, publication language.

#### 2.10.2 Network diagram and contribution matrix

We will present the network diagram for all the primary outcomes, which are the visualizations for the direct comparisons between the different TCM treatment methods and the routine pharmacotherapy groups. In these diagrams, nodes (circles) indicate TCM treatment methods, and their sizes are proportional to the sample size of respective intervention. Edges (lines) represent direct comparisons, the width of which is proportional to the number of available studies.

Each direct comparisons have different impacts on the results of NMAs, so it is necessary to understand which affect the most in the network. The contribution matrix will be used to describe the percentage contribution of each direct meta-analysis to the overall evidence. The operations above will be done by the Stata software (version 13.0; Stata Corporation, College Station, TX).

#### 2.10.3 Network meta-analysis

We will use WinBUGS 1.4.3 software (MRC Biostatistics Unit, Cambridge, UK) to carry out these NMAs. Under a Bayesian framework, we will use a Markov Chain Monte Carlo (MCMC) algorithm to perform both fixed and random effects estimates with vague priors for the 3 TCM treatment methods for each primary outcome. The achievement convergence will be assessed by trace plot, density plot (posterior distribution pattern of parameters, Bandwidth value and its mean with 95% CI) and Brooks Gelman Rubin diagnosis plot (shrink factor (mean, 97.5% CI) and PSRF value).^[32]^ The deviance information criterion (DIC) will be used to evaluate the fitting degree and complexity of the model, and the smaller the DIC, the better.^[33]^ The heterogeneity variance (τ^2^) estimated from the NMAs model will examine the statistical heterogeneity of global NMAs.^[34]^

We will use the surface under the cumulative ranking curve (SUCRA) value to obtain the comprehensive rank of the probabilities of the best TCM treatment method in each primary index.^[35]^ The SUCRA means that if an intervention always rank 1, the SUCRA is also 1; if keep last, then the SUCRA is 0, therefore, a higher SUCRA value indicates a better effect.^[36]^ The summary for all pairwise comparisons will be presented in a league table, as well as the probabilities of ranking the TCM treatment methods. The result diagram will be generated by Stata software. If the data are unavailable for quantitative analysis, we will summarize the evidence and conduct a descriptive analysis.

#### 2.10.4 Assessment of inconsistency

Investigating inconsistency will be not required in this network analysis because there is no direct comparative evidence among the three TCM treatment methods included in this review.

#### 2.10.5 Sensitivity analysis

Different levels of the methodological quality of trails may tend to affect the overall effects. We will employ the way of eliminating low-quality literature for sensitivity analysis to explore the stability conclusion, based on the results of RoB assessment, if any.^[37]^

#### 2.10.6 Publication bias

Egger regression test will be used for continuous data, and Peterʼs for dichotomies data to assess the publication bias, if the studies included are sufficient (n ≥10). If feasible, we will also employ the statistical model, suggested by Chaimani,^[38]^ to explore the possible existence of small-study effects in the network.

#### 2.10.7 Rating the confidence in estimates of the effect in NMA

The quality of evidences for each primary outcome will be assessed separately by 2 reviewers (PLJ and LXD) using the GRADE Working Group approach.^[39,40]^ The confidence in estimates will be categorized into 4 levels: high quality, moderate quality, low quality, and very low quality. Before the specific rating process, the 2 reviewers need to ensure the agreement on the understanding of rating standards. The flow of quality assessment involves following 4 steps:

Present the direct and indirect effect estimates,

Rate the quality of evidence from direct and indirect comparisons,

Present the NMA estimate for each comparison of the evidence network,

Rate the quality of evidence of the NMA effect estimates.

The content of rating the confidence for direct comparisons includes 5 items: risk of bias, indirectness, inconsistency, imprecision, publication bias. For indirect estimates, except the intransitivity assessment, the quality of evidences are based on the lower rating of the 2 pairwise estimates that contributes to the indirect estimate of the comparison of interest.

### 2.11 Ethics and dissemination

This systematic review will not require ethical approval because there are no data used in our study that are linked to individual patient data. In addition, findings will be disseminated through peer-review publications.

## 3 Discussion

CHFs have been used in China for thousands of years and are widely used as complementary and alternative therapies in the world. The mechanism of IPF is complex and multiple, and sufficient evidence have shown it is closely associated with immunity and inflammation. A host of formulas or extracts of herb ingredients have been confirmed the effects on regulating cytokines, signal transduction pathways, and oxidative stress and inhibiting extracellular matrix synthesis.^[41–43]^ Due to the diversity of active ingredients in CHFs and the potential synergistic effect among them, which can make them have a wide range of targets and multiple therapeutic mechanisms.

There are many modern drugs combined with CHFs to treat for IPF and the TCM treatment method reflection in most of which can be classified into 3 categories, which are “activating blood circulation and removing blood stasis” method, “tonifying Qi and activating blood circulation” method and “dredging collaterals” method. All the 3 TCM treatment method are devoting to alleviate the main symptoms of cases. Although the composition of the specific herbs in CHFs is slightly different under the guidance of the same TCM treatment method, the main herbs in formulas belong to the same category, and the focus of treatment is similar. Therefore, it is necessary to obtain the relative advantages among different TCM treatment method, and we hope this systematic review will provide more reliable evidence for clinical decision-making.

## Author contributions

Pang Lijian and Lv Xiaodong conceived and designed this protocol. Pang Lijian drafted this article. Liu Jian-Ping reviewed and edited this article.

**Conceptualization:** Lijian Pang.

**Funding acquisition:** Lijian Pang.

**Methodology:** Lijian Pang, Jian-Ping Liu.

**Software:** Lijian Pang.

**Writing – original draft:** Lijian Pang.

**Writing – review & editing:** Jian-Ping Liu, Xiaodong Lv.
